# O092: Clean hands: an university extension project

**DOI:** 10.1186/2047-2994-2-S1-O92

**Published:** 2013-06-20

**Authors:** AFV Tipple, JLU Spagnoli, ZCP Neves, JEM Santos, FCR Cesar, JPDA Trindade, KCDO Batista

**Affiliations:** 1 Faculdade de Enfermagem, Universidade Federal de Goiás, Goiânia, Brazil

## 

This video presents the story of a university extension project called "Clean Hands" which originated from a master's dissertation in 2006, based at the Center for Studies and Nursing Research for the Prevention and Control of Healthcare Associated Infections (NEPIH) based at the Center for Studies and Nursing Research for the Prevention and Control of Healthcare Associated Infections (NEPIH) at the School of Nursing, Federal University of Goiás, Brazil, and is registered with the Pro-Chancellor of Extension at the university. Since its creation, the project has developed activities to encourage the establishment of hand hygiene (HH) in health care, in cooperation with professionals, academics, patients and caregivers, as well as in scientific events, with academics and health professionals. After the outbreak of H1N1 in 2009, the project developed campaigns in municipal daycare centers (CMEI), the Municipal Department of Education, of the city of Goiânia, Goiás, Brazil, targeting children, parents and workers. In these campaigns, different promotion strategies are used: informative stylized banners depicting HH; educational brochures, a song parody CD, demonstration of proper HH technique, using poster paints on children’s hands; a puppet theater; and face-to-face discussions about the importance of, obstacles to, and benefits of HH. Annually, the project hosts a festival of parodies with the theme of HH, called "CANTA FEN", which brings together academics and healthcare professionals. The project’s day-to-day operations are normally run by five students, supported by the other members of NEPIH, currently 33 staff members (faculty, undergraduate and graduate), participating in the activities of the project. Up to February 2013, the project has performed about 180 campaigns (45 were for children) reaching approximately 8,000 people. Participation in the project has contributed to the development of skills and competencies with regard to the implementation of health promotion strategies with different audiences and requires that students constantly stay up to date on the subject. The festival of parodies has helped to empower its members to conduct scientific and cultural events and promote emphasis of the subject in a playful manner.

## Disclosure of interest

None declared.

